# Detection of *Bartonella* spp. in farmed deer (Artiodactyla: Cervidae) using multiplex assays in the Qinghai-Tibet Plateau, China

**DOI:** 10.1128/spectrum.04120-23

**Published:** 2024-05-24

**Authors:** Yu Miao, Wentao Guo, Wen Zhang, Zhizheng Chen, Delan Mian, Ruixiao Li, Ailing Xu, Min Chen, Dongmei Li

**Affiliations:** 1 National Key Laboratory of Intelligent Tracking and Forecasting for Infectious Diseases, National Institute for Communicable Disease Control and Prevention, Chinese Center for Disease Control and Prevention, Beijing, China; 2 Qinghai Provincial Institute of Endemic Disease Control and Prevention, Xining, China; 3 Qilian County Center for Disease Control and Prevention, Haibei Tibetan Autonomous Prefecture, Qilian, China; 4 School of Public Health, Cheeloo College of Medicine, Shandong University, Jinan, China; Texas A&M University, College Station, Texas, USA

**Keywords:** *Bartonella*, ruminant, qPCR, Nanopore sequence, phylogenetic analysis

## Abstract

In this study, we investigated the prevalence of *Bartonella* in deer from Qilian County, Qinghai Province, China. Blood samples were collected from 69 red deer, 40 white-lipped deer, and 27 sika deer. The detection of *Bartonella* spp. has been conducted. The overall prevalence of *Bartonella* was 33.6% (46/135). Species-specific prevalence was 50.72% in red deer (35/69), 20.00% in white-lipped deer (8/40), and 11.11% in sika deer (3/27). There were significant differences in the prevalence rates among the different species of deer. The amplicon sequence comparison revealed a high homology of the ruminant-associated *Bartonella* spp. Nanopore sequencing further confirmed the results. *Bartonella* reads were presented in each of the qPCR-positive samples. Phylogenetic analysis indicated that the *Bartonella* sequences detected in deer blood were closely related to ruminant-borne *Bartonella* spp. In summary, we reported the *Bartonella* prevalence of different deer species in Qinghai, and there were at least one species of ruminant-associated *Bartonella*, *B. schoenbuchensis*.

**IMPORTANCE:**

This is the first report about *Bartonella* infections in the deer population from China. We found that there were two species of *Bartonella* and an unidentified species of *Bartonella* among the unculturing strains carried by these deer populations. We first used Nanopore sequencing to detect *Bartonella* from deer blood samples and indicated that Nanopore sequencing is beneficial to detect pathogens due to its advantage of real-time and high sensitivity.

## INTRODUCTION

*Bartonella* is a group of Gram-negative intracellular facultative aerobic bacteria. More than 39 validated species and three subspecies of *Bartonella* have been isolated as of 12 November 2023 (http://www.bacterio.net/bartonella.html), while more than 54 species are not validly published. *Bartonella* can parasitize the red blood cells of mammals. Mammals such as Rodentia, Chiroptera, Lagomorpha, Artiodactyla, Carnivora, Primates, etc. have been reported to carry *Bartonella*. It’s also been reported that turtles carry *Bartonella* (1–3). It can be transmitted within the population via blood-sucking arthropods, for example, body lice, fleas, biting flies, and deer keds, and by biting or scratching by reservoir mammals (such as cats and dogs) (4, 5).

Hosts infected with *Bartonella* spp. in nature are asymptomatic. However, some species of *Bartonella* spp. can cause diseases in humans and mammals, hence being recognized as emerging infectious diseases (6). For example, *B. henselae* can cause cat-scratch disease who are scratched or bitten by cats and dogs (7), while *B. bacilliformis* can cause Carrion disease through sandfly bites (8). *B. quintana* is the pathogen of trench fever (9). The main clinical symptoms of bartonellosis include fever, lymphadenopathy, bacteremia, endocarditis, bacillary angiomatosis, and peliosis hepatitis (10–13).

Deer (Artiodactyla: Cervidae) are ruminants that can be infected with ruminant-associated *Bartonella* species, including *B. schoenbuchensis*, *B. capreoli*, and *B. bovis* (14). Among other ruminants, cattle can be infected with *B. bovis* and *B. chomelii*, while sheep can be infected with *B. melophagi* (15, 16). Deer ked (*Lipoptena cervi*) and other blood-sucking vector arthropods transmit *Bartonella* into the deer population (17). When an adult deer ked attaches to a host infected with *Bartonella*, it sucks blood and lays larvae. Through vertical transmission from the mother to its progeny, the larvae will carry *Bartonella*. After pupation, *Bartonella* is transmitted from the pupa to the adult. The adult deer keds will find other suitable hosts (18). *B. schoenbuchensis* is the leading cause of human deer ked dermatitis, which has similar clinical symptoms to cat-scratch disease (19, 20). Unlike cat-scratch disease, deer ked dermatitis does not usually present with lymphadenopathy. Within 6–24 h after a deer ked bite, dermatitis consisting of a few to 20–50 red papules varying in size from a few millimeters to 1–2 cm appears primarily on the scalp, neck, and upper back. These symptoms usually subside in several weeks (21). The delayed-type hypersensitivity skin test is positive when whole deer ked extracts are used for the skin test (22).

Blood culture is now considered the gold standard for detecting pathogens in blood. However, this method has some limitations because it is time-consuming and can only identify microorganisms that grow under specific culture conditions (23). Over the past few years, the molecular detection of bacterial pathogens has become increasingly sophisticated (24). Quantitative real-time polymerase chain reaction (qPCR) has the characteristics of high sensitivity, specificity, and accuracy, which are suitable for early diagnosis of infection. Therefore, the current main detection method of *Bartonella* is qPCR (25). Recently high-throughput sequencing technology has been applied to detect pathogens from clinical samples (26). Nanopore sequencing, as a novel third-generation sequencing, has the characteristics of long read fragment and short detection time (27–30), providing direct and real-time detection and identification of pathogens in animal tissue samples (31).

There have been some reports of deer and their parasites infected with *Bartonella* in some countries and regions, but there are no reports in China. To explore the infection rate of *Bartonella* spp. in the deer population in China initially, we selected a sampling point to perform this study. In this study, we used multi-test methods including qPCR, PCR, and Nanopore sequencing to explore the prevalence of *Bartonella* spp. in deer from the Qinghai-Tibet plateau, China.

## MATERIALS AND METHODS

### Material collection

Qilian County (38°16′54" N, 99°51′11" E) is located in north-western China with an average altitude of about 2,962 m. Qilian County has diverse geographical landforms with unique original ecological landscapes such as grasslands, snow mountains, forest seas, canyons, and glaciers. Qinghai Province is an important natural habitat for deer.

A total of 136 deers were sampled from a ranch in Qilian County. Peripheral blood samples from 69 red deer (*Cervus canadensis*), 40 white-lipped deer (*Przewalskium albirostris*), and 27 sika deer (*Cervus nippon*) were collected in K3-EDTA anticoagulant tubes. Date, species, and sex were recorded. Samples were stored in a refrigerator at −80°C.

### *Bartonella* culture

Deer blood was diluted in Bacto Brain Heart Infusion liquid medium (Becton, Dickinson and Company, USA) in a proportion of 1:4. Then spread on a Difco Tryptic Soy Agar (TSA) plate (Becton, Dickinson and Company, USA) supplemented with 5% defibrinated sheep blood and incubated at 37°C with 5% CO_2_ for 28 days for primary bacterial isolation.

### DNA extraction

Use the Wizard Genomic DNA Purification Kit (Promega Corporation, USA) to extract DNA from deer blood samples. The concentration of the total DNA was determined with NanoDrop-1000 Lite Spectrophotometer (ND-1000, Thermo Fisher Scientific, USA) and Qubit 4.0 Fluorometer (Invitrogen, Thermo Fisher Scientific, USA) and stored at −40°C.

### PCR detecting and sequencing

DNA extracted from the individual blood samples was subjected to qPCR for *Bartonella* spp. detection targeting a region in the *ssrA* gene. Nucleotide primers used in this study are listed in Table 1. DNA of *Bartonella henselae* (ATCC 49882) and water served as positive and negative controls. The qPCR amplifications were performed in 20 µL reaction mixtures containing 10 µL 1× Taq buffer Mix, 0.8 µL for each forward and reverse primer (ssrA-F–ssrA-R), 0.8 µL fluorescence probe (ssrA-T), and 3 µL DNA template, complete with deionized water (32). Each qPCR was carried out in a real-time fluorescence ratio PCR instrument (Bio-Rad, USA) with the following amplification conditions: initial denaturation at 95°C for 3 min; followed by 40 cycles of amplification at 95°C for 15 s, 60°C for 45 s. Every DNA sample was conducted in two parallel experiments. Samples were considered positive when their Ct value was less than 40.

**TABLE 1:** Oligonucleotide primers and probes used in this study

Target gene	Primer	Primer direction	Primer sequence (5′−3′)	Product length (bp)
*ssrA* ^*a*^	ssrA-F	Forward	GCTATGGTAATAAATGGACAATGAAATAA	300
	ssrA-R	Reverse	GCTTCTGTTGCCAGGTG	
	ssrA-T	Probe	FAM-ACCCCGCTTAAACCTGCGACG-BHQ1	
*gltA* ^*b*^	BhCS.781p	Forward	GGGGACCAGCTCATGGTGG	379
	BhCS.1137n	Reverse	AATGCAAAAAGAACAGTAAACA	
16S rRNA^*c*^	27F	Forward	AGAGTTTGATCMTGGCTCAG	1,500
	1492R	Reverse	GGTTACCTTGTTACGACTT	

^*a*^ qPCR.

^*b*^ Conventional PCR.

^*c*^ Conventional PCR for preparing Nanopore sequence libraries.

To further analyze whether the positive samples were from ruminant-associated *Bartonella*, conventional PCR was used to generate amplicons of the housekeeping gene for Citrate synthase (*gltA*). The conventional PCR amplifications for the *gltA* gene were performed in 25 µL reaction mixtures containing 12.5 µL 2× EasyTaq PCR Supermix for Page (TransGen Biotech, Beijing, China), 1 µL for each forward and reverse primers (BhCS.781p–BhCS.1137n) and 3 µL DNA template, complete with deionized water (33). The conventional PCR was carried out in PCR LabCycler (Standard P, Sensoquest, Germany) with the following amplification conditions: initial denaturation at 94°C for 2 min; followed by 30 cycles of denaturation at 94°C for 30 s, annealing at 50°C for 30 s, and extension at 72°C for 2 min; followed by a final extension at 72°C for 5 min (34).

Due to the low yield of amplicons, which were not sufficient for Sanger sequencing, the secondary PCR amplification was performed targeting the *gltA* genes. Templates were the conventional PCR products of the *gltA* gene. To further verify that the sequences detected are ruminant-associated *Bartonella*, the qPCR products of the *ssrA* gene were used as templates for secondary conventional PCR amplification targeting the *ssrA* gene. The secondary PCR amplifications use the same reaction parameters and primers as the first time. The products of secondary PCR amplification were visualized by electrophoresis on gels with 1% agarose by checking the gels under UV light. The amplicon for the *gltA* gene fragment is 379 bp, while the *ssrA* gene fragment is over 300 bp. Verified fragments of gene *ssrA* and *gltA* were sequenced by Sangon Biotechnology Corporation (Shanghai, China). Alignment sequences obtained from sequencing were blasted on the NCBI website by using BLASTn (Basic Local Alignment Search Tool, https://blast.ncbi.nlm.nih.gov).

### Nanopore detection

The 16S Rapid Amplicon Barcoding Kit (Oxford Nanopore Technologies, ONT, Oxford, UK, SQK-RAB204) was used to prepare the 16S rRNA gene sequence libraries following the standard procedures described by ONT. The amplifications were conducted in 50 µL reaction volume, including 10 µL DNA, 25 µL LongAmp Taq 2× master mix (New England Biolabs, Ipswich, MA, USA), 1 µL 16S barcoded Nanopore sequence primers (14F–1492R in Table 1) and 14 µL ddH2O, under the program of one denaturation cycle at 95°C for 1 min, followed by 25 cycles of amplification at 95°C for 20 s, 55°C for 30 s, 65°C for 2 min and a final extension step at 65°C for 5 min (35).

The PCR amplification product was purified by adding 30 µL magnetic beads (Agency AMPure XP Reagent) mixing and incubating at room temperature for 5 min, washing the mixture with the 70% ethanol solution twice, adding 3 µL Tris-HCl (pH 8.0, containing 50 mmol/L NaCl) after drying in the air. The supernatant contains the purified nucleic acid. To sequence, pool equal amounts of amplicons per sample, and incubate with Library Loading Beads (Oxford Nanopore Technologies, ONT, Oxford, UK). MinKNOW (MinION software, version 23.04.3, Oxford Nanopore Technologies, ONT, Oxford, UK) was used to control sequencing devices by connecting to the sequencing device on the computer. This software performs several core tasks, including data acquisition, real-time analysis, basecalling, and data streaming. Check the flow cell before loading the DNA library to assess the number of pores available, the number of active pores should be over 800. Then, load the library onto the MinIon flow cell (version R9.2, Oxford Nanopore Technologies, ONT, Oxford, UK) in the MinIon Nanopore sequencer (Oxford Nanopore Technologies, ONT, Oxford, UK). Set up the corresponding reagent kit and parameters on the computer. The duration of the sequencing experiment was set up to 24 h. During the sequencing experiment, the sequencing device can generate raw data. MinKNOW takes the raw data and converts it into reads by recognizing the distinctive change in current across the flow cell membrane that occurs when a DNA strand enters and leaves the pore, then basecalls the reads and writes out the data into files. The Metrichor Ltd. analysis platform EPI2ME (version 3.6.2) was used to generate and analyze the basecalling of Nanopore signals. The cutoff of reads number of Nanopore sequencing is 10.

### Phylogenetic analysis

The Maximum-likelihood (ML) phylogenetic trees were constructed by *ssrA* and *gltA* gene sequences, respectively. Sequences used to construct phylogenetic trees include the sequences of the *ssrA* and *gltA* genes obtained from the samples of deer used to construct phylogenetic trees, the other known as *Bartonella* spp. sequences and all the *Bartonella* strains of the *gltA* gene which were isolated from ruminants were downloaded from the NCBI website (https://www.ncbi.nlm.nih.gov). The sequence alignment was carried out using the ClusterW tool in the MEGA program (v 11). ML phylogenetic trees were constructed using iqtree (version 1.6.12) with 1,000 bootstrap replicates. The *ssrA* sequence of *Brucella melitensis* (accession number CP044985) and the *gltA* sequence of *Brucella abortus* (accession number NC_006932.1) were employed as out-group sequences. The generated phylogenetic trees were displayed and annotated in the iTOL: Interactive Tree of Life website (https://itol.embl.de).

### Statistical analysis

R studio software (v 3.5) was used for statistical analysis. Chi-square and Fisher’s exact tests were used to evaluate the differences in infection rates among the tested population. *P*＜0.05 was considered statistically significant.

### Nucleotide sequence accession numbers

Sequences of the uncultured *Bartonella* gene fragments generated in this study were deposited in the NCBI GenBank database under accession numbers PP436807 and PP436808 for the *gltA* gene, PP436784 to PP436806, PP529449 and PP529450 for *ssrA* gene.

## RESULTS

### Bartonella culture

Attempted to isolate *Bartonella* from the deer blood samples ended up failing after culturing for 28 days. Except for a few medium plates, most of the plates were contaminated by miscellaneous bacteria. These included *Cellvibrio fibrivorans*, *Cutibacterium acnes*, and some other bacteria.

### Prevalence of *Bartonella* spp. in deers

qPCR test results are shown in Table 2. The prevalence of *Bartonella* spp. in all deer samples was 33.8% (46/136). Among them, 35 of 69 (50.72%) red deer, 8 of 40 (20.00%) white-lipped deer, and 3 of 27 (11.11%) sika deer were detected positive. There were significant variations in infection rates (*P*＜0.05, Fisher’s exact test) among these three species. A total of 23 samples with cycle threshold (Ct) values were strongly positive (25＜Ct＜35), including 21 red deer samples and 2 white-lipped deer samples, which may indicate higher bacteremia in the deer. In addition, weak positive signals (Ct＞35) were detected in 23 samples, including 14 red deer samples, 6 white-lipped deer samples, and 3 sika deer samples.

**TABLE 2:** The results for qPCR

		Number of detection	Positive number	Strong positive number	Weak positive number	Positive rate (%)
Species	Red deer	69	35	21	14	50.72
	White-lipped deer	40	8	2	6	20.00
	Sika deer	27	3	0	3	11.11
Total		136	46	23	23	33.80

The sequences of the *ssrA* gene from 25 samples and the sequences of the *gltA* gene from 4 of these 25 samples were successfully sequenced and alignment after the secondary PCR amplification. The BLAST results are shown in Table 3. Twenty four of these 25 samples were from red deer, and one of them was from white-lipped deer. All of these 25 samples were detected positive by qPCR. Two of 4 sequences of the *gltA* gene were blasted to *Bartonella schoenbuchensis*, others were blasted to *B. tribocorum* and *B. krasnovii* with lower percent identity (less than 96%), respectively. All these 25 sequences of the *ssrA* gene were blasted to ruminant-associated *Bartonella* (Table 4), but there is little difference in percent identity between ruminant-associated *Bartonella* (less than 1%). Therefore, the BLAST results of the *ssrA* gene and the *gltA* gene can't identify species, but they can verify that these sequences are ruminant-associated *Bartonella*.

**TABLE 3:** BLAST results of PCR amplification^*a*^

Sample no.	Host species	Optimal matching of *gltA* gene fragment	Percent identity of *gltA* (%)	Optimal matching of *ssrA* gene fragment	Percent identity of *ssrA* (%)
2	Red deer	*B. schoenbuchensis*	97.44	*B. melophagi*	99.60
3	Red deer	*B. tribocorum*	91.83	*B. chomelii*	99.13
4	Red deer	*B. krasnovii*	94.54	*B. chomelii*	99.19
11	Red deer	–	–	*B. bovis*	98.81
12	Red deer	–	–	*B. chomelii*	99.13
17	Red deer	–	–	*B. chomelii*	100.00
28	Red deer	–	–	*B. melophagi*	99.21
30	Red deer	–	–	*B. chomelii*	100.00
31	Red deer	*B. schoenbuchensis*	98.01	*B. bovis*	98.80
32	Red deer	–	–	*B. chomelii*	100.00
37	Red deer	–	–	*B. chomelii*	100.00
41	Red deer	–	–	*B. chomelii*	98.28
44	Red deer	–	–	*B. melophagi*	99.60
45	Red deer	–	–	*B. bovis*	98.41
121	Red deer	–	–	*B. melophagi*	98.81
122	Red deer	–	–	*B. bovis*	98.41
126	Red deer	–	–	*B. chomelii*	100.00
127	Red deer	–	–	*B. chomelii*	96.12
128	Red deer	–	–	*B. bovis*	95.67
129	Red deer	–	–	*B. melophagi*	98.81
130	Red deer	–	–	*B. chomelii*	98.41
131	Red deer	–	–	*B. chomelii*	92.83
132	Red deer	–	–	*B. bovis*	99.21
133	Red deer	–	–	*B. chomelii*	97.18
136	White-lipped deer	–	–	*B. melophagi*	99.66

^*a*^ "–" means there are no experimental results.

**TABLE 4:** BLAST results of PCR amplification of the ssrA gene

Sample no.	Host species	Percent identity of *B. melophagi* (%)	Percent identity of *B. chomelii* (%)	Percent identity of *B. bovis* (%)	Percent identity of *B. schoenbuchensis* (%)	Percent identity of *B. capreoli* (%)
2	Red deer	99.60	98.81	98.80	98.41	97.22
3	Red deer	98.27	99.13	98.25	98.70	97.39
4	Red deer	98.38	99.19	98.39	98.78	97.56
11	Red deer	98.39	98.39	98.81	97.99	96.79
12	Red deer	98.26	99.13	98.28	98.69	97.38
17	Red deer	99.21	100.00	99.20	99.60	98.41
28	Red deer	99.21	98.41	98.39	98.02	96.83
30	Red deer	99.21	100.00	97.81	99.60	98.41
31	Red deer	98.02	98.02	98.80	97.62	96.43
32	Red deer	99.21	100.00	99.20	99.60	98.41
37	Red deer	99.21	100.00	99.20	99.60	98.41
41	Red deer	97.44	98.28	97.46	97.85	96.57
44	Red deer	99.60	98.81	98.80	98.41	97.22
45	Red deer	96.79	96.79	98.41	96.39	95.18
121	Red deer	98.81	98.41	98.39	98.02	96.83
122	Red deer	96.79	96.79	98.41	96.39	95.18
126	Red deer	99.33	100.00	98.61	98.99	98.41
127	Red deer	95.17	96.12	95.63	95.63	95.15
128	Red deer	94.90	94.90	95.67	94.51	94.07
129	Red deer	98.81	98.41	98.39	98.02	96.83
130	Red deer	97.62	98.41	97.59	98.01	96.81
131	Red deer	92.06	92.83	92.83	92.43	91.24
132	Red deer	97.99	97.99	99.21	97.59	96.39
133	Red deer	96.39	97.18	96.80	96.77	95.56
136	White-lipped deer	99.66	98.81	98.26	97.99	97.22

### Nanopore sequencing

The sequencing library was constructed by the purified PCR product of 16S rRNA from 46 DNA samples of deer blood which were positively detected by qPCR. After sequencing on the MinIon sequencer for 24 h, a total of 1028.5 Mbases of data were generated from every barcode with 669,215 reads. The average sequence length is 1,532 bp. All the deer blood samples that were positive tested by qPCR had reads aligned to *Bartonella*. There are many more reads of *B. schoenbuchensis* R1 than other *Bartonella* spp. in every sample barcode (Table 5).

**TABLE 5:** Results of Nanopore sequencing

Sample no.	Nanopore total reads	*Bartonella* reads	*B. schoenbuchensis* reads	*B. capreoli* reads	*B. bovis* reads	Anaplasmataceae family reads	*Anaplasma* reads	*Ehrlichia* reads
2	20,029	1,778	1,695	32	4	14,177	44	50
3	2,604	94	77	3	2	5	0	0
4	150	10	10	0	0	5	0	0
11	84,952	26,508	25,382	415	69	44,440	162	162
12	2,698	75	73	1	0	2,238	8	8
13	1,767	83	77	3	0	426	3	2
16	3,108	288	277	4	0	1,198	8	2
17	16,577	354	341	5	2	13,777	48	45
18	34,607	1,275	1,224	22	4	23,925	79	63
19	8,043	540	512	9	3	5,341	17	23
20	14,371	280	267	6	1	2,022	4	9
22	79,843	4,278	4,129	51	9	63,025	223	240
23	19,329	62	59	1	1	1,367	6	5
25	55,798	290	282	4	1	3,306	8	11
28	16,778	758	729	11	1	12,668	45	34
30	4,764	403	392	4	0	2,255	8	10
31	12,476	3,178	3,018	54	10	7,615	24	33
32	11,957	407	370	7	0	7,728	35	30
35	1,581	142	138	3	0	404	0	0
37	12,326	1,140	1,087	17	2	8,178	17	29
41	16,355	239	235	1	1	13,270	47	52
44	82,988	11,096	10,650	157	33	62,471	259	217
45	27,421	123	113	2	1	19,442	158	78
58	24,771	8,124	7,694	161	34	13,081	102	46
70	2,368	653	617	11	0	904	8	5
72	9,617	8,338	7,870	166	38	4	0	0
75	1,790	282	267	3	0	656	6	2
80	4,863	4,012	3,770	83	17	87	0	1
81	11,280	9,719	9,195	185	40	52	1	0
88	5,577	504	477	6	3	296	0	2
93	3,091	313	287	10	1	334	5	1
97	5,578	4,636	4,388	81	18	4	0	0
121	457	397	375	10	1	0	0	0
122	28,201	25,537	24,126	413	219	5	1	0
123	6,578	2,553	2,437	40	12	2	0	0
126	90	55	54	0	0	1	0	0
127	2,098	1,076	1,021	17	6	379	5	1
128	8,189	7,514	7,223	79	17	1	0	0
129	1,293	261	258	2	0	923	5	4
130	788	697	668	14	3	0	0	0
131	26	12	12	0	0	0	0	0
132	29	16	15	0	0	0	0	0
133	5,278	694	672	13	0	47	0	0
134	609	439	426	3	0	0	0	0
135	139	62	60	1	0	0	0	0
136	144	21	18	1	0	0	0	0

In addition, analyzing the Nanopore sequencing, the most abundant read among all bacterial reads was mapped to Anaplasmataceae (*N* = 326,059 reads), detected in 39 out of 46 samples (84.78%). Among them, 4,929 (1.51%) reads can map to *Anaplasma* or *Ehrlichia. Anaplasma phagocytophilum* was detected in 12 samples (28.09%), while *Ehrlichia ruminantium* in 9 samples (19.57%). Some other bacteria were also detected by Nanopore sequencing.

### Phylogenetic analysis

**Fig 1:**
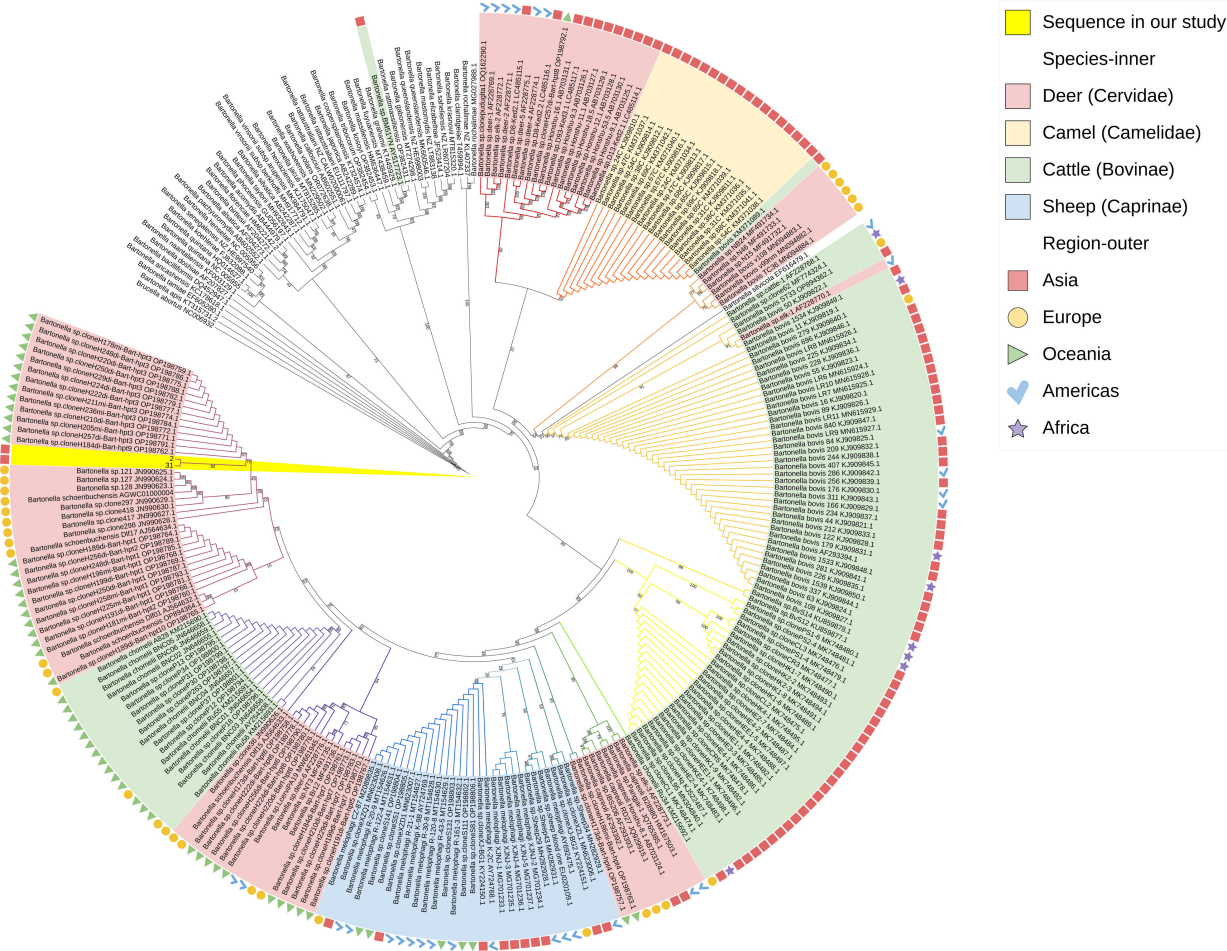
Maximum likelihood phylogenetic tree based on the *gltA* gene.

To further explore the relationship of the sequences from Chinese deer-associated *Bartonella* with globally recognized species of *Bartonella*, we performed a phylogenetic analysis based on the alignment of *gltA* and *ssrA* genes, respectively. The *gltA* phylogenetic tree was generated by 291 bp sequences (Fig. 1). All the ruminant-associated *Bartonella* sequences in this tree were clustered together and showed apparent host and partial spatial aggregation. The two sequences (2 and 31) we successfully assembled and the sequences of deer from Europe and Oceania were clustered together with the known species *B. schoenbuchensis*. In the BLAST result, the known species with the highest identity of both these two sequences was also *B. schoenbuchensis*. Most Europe and Oceania sequences of bovine were clustered together with the known species *B. chomeii*. Some European deer sequences were clustered with the known species *B. bovis*. The sequences associated with sheep from all places were clustered together with the known species *B. melophagi*. The sequences associated with camels from Israel were clustered together. Most deer sequences from Asia and the Americas were clustered together. Most cattle sequences from Asia, the Americas, and Africa were clustered with the known species *B. bovis*. Interestingly, some strains isolated from the same region but from different host species clustered together. For example, 18 Israeli isolates obtained from camels clustered with one Israeli isolate obtained from cattle (accession number KM371089.1). One isolate obtained from deer in the Americas (accession number AF228770.1) clusters with isolates obtained from cattle in the Americas.

**Fig 2:**
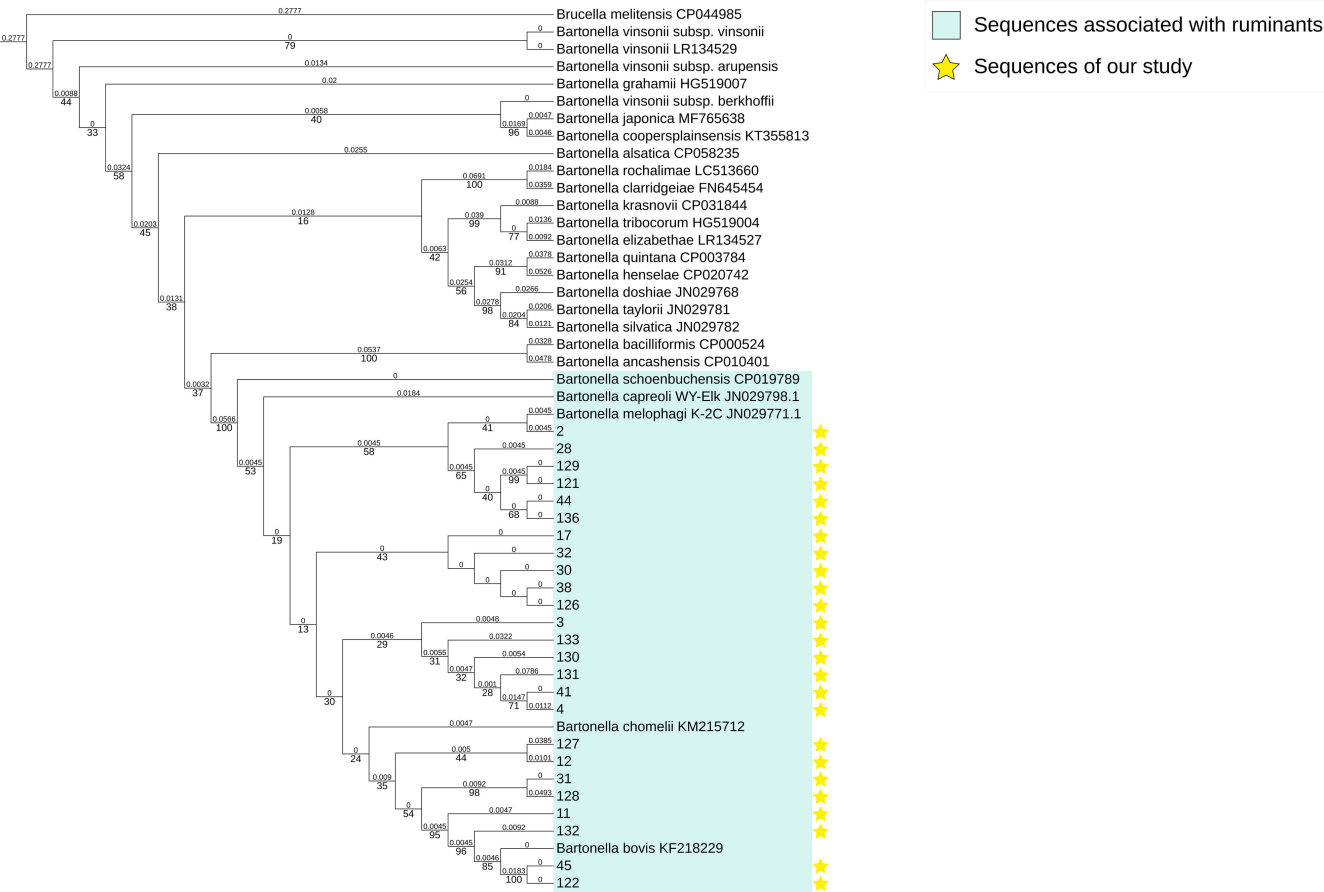
Maximum likelihood phylogenetic tree based on the *ssrA* gene.

In this study, we successfully sequenced and assembled 25 sequences of *ssrA* gene from deer blood samples. The *ssrA* gene phylogenetic tree was conducted based on the 261 bp sequences (Fig. 2). The sequences of our study were clustered in one clade, which included five known ruminant-borne *Bartonella* species. Among them are *B. schoenbuchensis*, *B. capreoli*, *B. melophagi*, *B. chomelii,* and *B. bovis*. The genetic distances are between 0 and 0.062 and the bootstrap values are low (less than 70). Therefore, the species cannot be identified. The maximum likelihood phylogenetic tree based on the *ssrA* gene can verify that the sequences of our samples are ruminant-associated *Bartonella* spp.

## DISCUSSION

*Bartonella* spp. are important zoonotic pathogens that have been isolated from peripheral blood red blood cells, spleen, kidneys, liver, and other tissues of many mammals (36). Many recent reports introduced the infection situation of *Bartonella* spp. among the deer population (37–41). In Malgorzata Adamska’s study, the prevalence of *Bartonella* DNA was 39% (23/59) in roe deer and 35% (7/20) in red deer, which were detected by blood and spleen samples in Poland (39). Cristina Perez Vera et al. researched the *Bartonella* spp. infection rates in moose in Finland. They found that *Bartonella* infection in blood samples of moose was 72.9% (108/148) (40). Carlos Sacristán et al. used PCR to detect the infection rate of *Bartonella* spp. in blood samples of 141 deers in Norway, including 65 moose, 41 red deer, and 35 reindeer. The prevalence of *Bartonella* DNA in moose was 75.4% (49/65), and in red deer was 4.9% (2/41). All the reindeer were tested negative (41). Christoph Dehio et al. collected 49 deer keds from seven roe deer and eight red deer in Germany to culture. Keds from seven out of eight roe deer (88%) and five out of seven red deer (71%) were culture-positive (22). Similar to the infection rate reported previously, the infection rate here was 50.7% in red deer samples, 20% in white-lipped deer, and 11.11% in sika deer, respectively, when detected by qPCR. The high infection rate of the deer population and the differences among the three species suggested that there may be potential vectors for transmission in the population, which may be species-specific.

*Bartonella* spp. has not been isolated in this study. *Bartonella* is difficult to culture and has strict quality requirements for samples. Low *Bartonella* load in deer blood is likely the main reason, as confirmed by the low Ct value of qPCR. Meanwhile, *Bartonella* spp. has an extended culture cycle, usually over 28 days. The growth rate of the other bacteria in the blood is faster than *Bartonella* spp. On the third day of cultivation, the miscellaneous bacterium was growing on the culture medium. When the colony of other bacteria covered the whole culture medium, *Bartonella* spp. could not culture.

*Bartonella* species were usually classified based on the *gltA* gene, a housekeeping gene of *Bartonella* (42). In the BLAST results, not all secondary PCR products have been successfully sequenced, mainly due to the low *Bartonella* DNA load in deer blood. Sequences with a percent identity of the *gltA* gene higher than 96% can be identified to this species (33), thus only two BLAST results of the *gltA* gene are credible. This may be due to the low sensitivity of the PCR detection of the *gltA* gene. The sequencing results of *gltA* and *ssrA* genes from the same sample were different, there can be several reasons. First, previous studies have shown that ruminant-associated *Bartonella* belongs to the *Bartonella bovis* species complex due to their high genetic similarity (43). The differences in identity between ruminant-associated *Bartonella* species are small, thus species cannot be identified. Based on the previous research (44), we believe that sequencing of *rpoB* and *groEL* genes should be performed for species identification. Secondly, the detection methods for these two genes are different. Templates for the secondary PCR amplification of the *gltA* gene were the product of conventional PCR amplification, but the templates for the second PCR of the *ssrA* gene were the product of qPCR amplification. The BLAST results confirm that these sequences are highly similar to ruminant-associated *Bartonella*.

In this study, we adopted Nanopore sequence technology on the qPCR-positive samples. The results of Nanopore sequencing can prove the result of qPCR. The sensitivity of Nanopore sequencing is higher evidenced by the *Bartonella* reads that were detected in samples with Ct values higher than 35 (up to 37–38). Furthermore, bacteria of the Anaplasmataceae family were detected in many deer samples. Some species of Anaplasmataceae are pathogenic to human beings, for example, *A. phagocytophilum* is the etiologic agent of human granulocytic anaplasmosis (45). The members of the Anaplasmataceae family carried by ruminants can be transmitted to humans or animals by arthropods such as ticks (46–48). *E. ruminantium*, which is associated with ruminants, was also detected in the deer samples by Nanopore sequencing, highlighting the advantage of this method. The Nanopore sequencing in our study was based on the 16 s rRNA gene, but the bacterium of the Anaplasmataceae family can’t be classified into species through this gene. Therefore, even though there were a large number of reads of the Anaplasmataceae family, they were not species-specific. Other pathogenic bacteria had been detected, which indicated that multiple opportunistic pathogens, such as *Bartonella*, *Anaplasma,* and *Serratia* are circulating in the deer in Qilian County.

Phylogenetic analysis revealed that the ruminant-associated *Bartonella* strains tend to be aggregated on host-based and partially spatial-based. The aggregation of strains from the same region in different hosts showed that the ruminant-associated *Bartonella* can be transmitted in different animal species. Ruminant-associated *Bartonella* are transmitted primarily by arthropod vectors. There have been some reports of the detection of *Bartonella* in deer keds and ticks (19, 20, 22, 37, 49). Regional specificity to some extent of the ruminant-associated *Bartonella* is likely.

The results of various methods of detection and analysis have proven that there was at least one species of ruminant-associated *Bartonella*, *B. schoenbuchensis*. However, our study still needs some improvement. We only have two *gltA* gene sequences successfully sequenced and aligned, thus we can't systematically describe the phylogenetic relationship between deer-borne *Bartonella* spp. from Qinghai and the global ruminant-associated *Bartonella*. We have successfully sequenced 25 *ssrA* gene sequences, but few ruminant-associated *ssrA* gene sequences are submitted to the NCBI website.

Deer ked is the most important ectoparasites of deer. It transmits *Bartonella* to humans by biting and blood-sucking resulting in deer ked dermatitis at the biting spot (22, 50). *B. bovis* is also one of the *Bartonella* spp. carried by ruminants including deer. Although no human infection case has been reported, *B. bovis* causes bovine endocarditis (49). Accordingly, it poses a threat to animal husbandry.

In conclusion, the presented data firstly show the presence of at least two different species of *Bartonella*, *B. schoenbuchensis,* and *B. bovis*, in populations of red deer, white-lipped deer, and sika deer in Qilian, China. The high prevalence of infection may indicate that transmitting arthropods exist in the population. A nationwide investigation is desired to better understand *Bartonella*’s prevalence in the deer population in China.
